# Physiotherapy and rehabilitation for survivors of female genital mutilation/cutting: a scoping review

**DOI:** 10.1186/s12905-026-04708-4

**Published:** 2026-07-18

**Authors:** Juliet Lucy Ekowa, Adaeze Imelda Onyekwelu, Kadiree Ekundayo Fatai

**Affiliations:** 1 Department of Physiotherapy, David Umahi Federal University of Health Sciences (DUFUHS), Uburu, Ebonyi State Nigeria; 2 Emerging Researchers and Professionals in Ageing–African Network (ERPAAN), Abuja, Nigeria; 3 International Institute for Sports Research Development and Rehabilitation, David Umahi Federal University of Health Sciences (DUFUHS), Uburu, Ebonyi State Nigeria

**Keywords:** Female genital mutilation/cutting, Physiotherapy, Rehabilitation, Pelvic health, Psychosexual rehabilitation, Chronic pelvic pain

## Abstract

**Background:**

Female genital mutilation/cutting (FGM/C) affects more than 230 million girls and women worldwide and is associated with chronic pelvic pain, pelvic floor dysfunction, lower urinary tract symptoms, psychosexual dysfunction, and reduced quality of life. Although physiotherapy and rehabilitation may contribute to multidisciplinary care, the available evidence has not been comprehensively synthesised.

**Objective:**

To map the existing evidence on physiotherapy and rehabilitation for women living with FGM/C and identify evidence gaps relevant to clinical practice and future research.

**Methods:**

A scoping review was conducted following the Arksey and O’Malley framework, Levac et al.‘s methodological enhancements, and PRISMA-ScR guidelines. Electronic databases and grey literature were searched from inception to January 2026. Eligible studies included adolescent (≥ 15 years) and adult women living with FGM/C, together with rehabilitation-relevant studies involving healthcare professionals. Data were charted and synthesised narratively.

**Results:**

Of 872 records identified, 21 studies met the inclusion criteria. Most evidence was observational or descriptive, with only one randomized controlled trial and one rehabilitation-focused case report directly evaluating rehabilitation interventions. Frequently reported rehabilitation-related impairments included psychosexual dysfunction, chronic pelvic pain, pelvic floor dysfunction, lower urinary tract symptoms, dyspareunia, and reduced quality of life. Reported rehabilitation approaches included pelvic floor rehabilitation, bladder retraining, biofeedback, psychosexual rehabilitation, and multidisciplinary care. Only one study included adolescents, and none specifically evaluated rehabilitation interventions for this population.

**Conclusion:**

Women living with FGM/C experience substantial rehabilitation needs affecting physical, psychosexual, urinary, and psychosocial health. Although physiotherapy and rehabilitation are increasingly recognised as important components of multidisciplinary care, direct evidence evaluating rehabilitation interventions remains limited. Future research should prioritise rehabilitation-focused intervention studies, implementation research, and standardised outcome measures to strengthen the evidence base and inform comprehensive, survivor-centred care.

**Supplementary Information:**

The online version contains supplementary material available at https://doi.org/10.1186/s12905-026-04708-4.

## Introduction

Female genital mutilation/cutting (FGM/C) remains a major global public health and human rights concern, affecting more than 230 million girls and women worldwide [1, 2]. FGM/C is defined as all procedures involving the partial or total removal of, or injury to, the female external genitalia for non-medical reasons [1]. The World Health Organization (WHO) classifies FGM/C into four major types according to the extent of anatomical alteration. Type I involves partial or total removal of the clitoral glans and/or prepuce (clitoridectomy), Type II involves partial or total removal of the clitoral glans and labia minora with or without excision of the labia majora (excision), Type III (infibulation) involves narrowing of the vaginal opening through the creation of a covering seal by cutting and repositioning the labia, with or without removal of the clitoral glans, while Type IV includes all other harmful procedures to the female genitalia performed for non-medical purposes, such as pricking, piercing, scraping or cauterisation [1]. The distribution of FGM/C types varies considerably across countries, regions and cultural groups [1–3].

Although complications have been reported across all FGM/C types, women with Type III FGM/C are generally reported to experience a greater burden of urogynaecological complications, pelvic floor dysfunction, chronic pain and psychosexual difficulties because of the more extensive anatomical alteration and scar formation [1, 4–8]. Nevertheless, rehabilitation needs are not limited to Type III FGM/C, and survivors across all WHO FGM/C types may experience functional impairments requiring physiotherapy and multidisciplinary rehabilitation [1, 4–6, 9–14].

The consequences of FGM/C extend far beyond the initial procedure and encompass physical, psychological, sexual and social dimensions of health. Acute complications include severe pain, haemorrhage, infection, urinary retention and genital trauma, whereas long-term sequelae include chronic pelvic pain, dyspareunia, lower urinary tract symptoms, urinary and faecal incontinence, pelvic organ prolapse, vulvar scarring, epidermal cysts, neuromas, impaired sexual function, altered body image, emotional distress, anxiety and reduced quality of life [1, 4–6, 9–14]. These adverse outcomes are frequently compounded by stigma, cultural barriers and limited access to culturally responsive healthcare services, further contributing to long-term disability and reduced participation in daily life [11, 14].

The long-term consequences of FGM/C can be understood using the International Classification of Functioning, Disability and Health (ICF), which conceptualises disability as the interaction between impairments, activity limitations, participation restrictions and contextual factors [15]. Within this framework, chronic pain, urinary dysfunction, pelvic floor disorders and psychosexual difficulties may adversely affect intimate relationships, employment, social participation and overall wellbeing. Consequently, effective management extends beyond medical or surgical care and requires interventions that promote functional recovery, participation and quality of life.

Pelvic floor dysfunction, lower urinary tract symptoms, chronic pelvic pain and dyspareunia are among the most common FGM/C-related functional impairments that may benefit from physiotherapy and rehabilitation [4–7, 9–12]. Physiotherapists have an important role within multidisciplinary rehabilitation through evidence-informed assessment, education and interventions targeting pelvic floor muscle dysfunction, chronic pain, scar-related tissue restrictions, bladder dysfunction, psychosexual dysfunction and functional limitations [1, 16–20]. Although many rehabilitation principles used in pelvic health physiotherapy are likely to be applicable to women living with FGM/C, management may require additional consideration of scar-related tissue restriction, altered genital anatomy, trauma-informed care and culturally responsive practice. In broader women’s health populations, interventions such as pelvic floor muscle training, biofeedback, bladder retraining, manual therapy and multidisciplinary rehabilitation have demonstrated benefits for pelvic floor dysfunction, chronic pelvic pain, urinary symptoms and dyspareunia [16–20]. However, the extent to which these interventions are applicable and effective for survivors of FGM/C remains uncertain.

Despite increasing recognition of the rehabilitation needs of women living with FGM/C, the evidence relating specifically to physiotherapy and rehabilitation remains fragmented. Existing literature has predominantly focused on symptom burden, survivor experiences, healthcare needs and multidisciplinary management, whereas direct evaluation of physiotherapy interventions and rehabilitation outcomes remains scarce [7, 10, 21, 22]. Previous reviews have examined broader healthcare provision, psychosexual outcomes, incontinence, fistula rehabilitation and the wider health consequences of FGM/C [2, 10, 23–25]. However, none has comprehensively synthesised physiotherapy interventions, rehabilitation pathways, rehabilitation outcome measures or the role of physiotherapy within multidisciplinary FGM/C care. Consequently, important knowledge gaps remain regarding rehabilitation practice, service delivery and priorities for future research.

A comprehensive synthesis of the available evidence is particularly important given the increasing emphasis on survivor-centred, culturally responsive and trauma-informed care [1, 2, 14, 26]. Furthermore, the heterogeneous and emerging nature of rehabilitation research makes a scoping review the most appropriate methodology for mapping the breadth, characteristics and knowledge gaps within the existing literature [27–29].

Therefore, this scoping review aimed to systematically map and characterise the existing evidence on physiotherapy and rehabilitation for survivors of FGM/C. Specifically, the review sought to identify FGM/C-related functional impairments relevant to rehabilitation, describe physiotherapy interventions and rehabilitation pathways, examine rehabilitation outcome measures, and identify evidence gaps to inform clinical practice, policy and future research.

## Methodology

### Study design

This scoping review was conducted in accordance with the methodological framework originally proposed by Arksey and O’Malley [27], refined by Levac et al. [28], and informed by current Joanna Briggs Institute (JBI) guidance for scoping reviews. The review was reported in accordance with the Preferred Reporting Items for Systematic Reviews and Meta-Analyses Extension for Scoping Reviews (PRISMA-ScR) guidelines [29]. A scoping review methodology was considered appropriate because evidence relating to physiotherapy and rehabilitation for survivors of female genital mutilation/cutting (FGM/C) was anticipated to be heterogeneous in terms of study design, methodology, and disciplinary focus. The review protocol was developed a priori and prospectively registered with the Open Science Framework (OSF; DOI: https://doi.org/10.17605/OSF.IO/XW9G4) to enhance methodological transparency and reproducibility. A completed PRISMA-ScR checklist is provided in the supplementary materials.

### Identifying relevant studies

A comprehensive search strategy was developed using the Population–Concept–Context (PCC) framework to identify evidence relating to physiotherapy and rehabilitation for survivors of female genital mutilation/cutting (FGM/C). Both controlled vocabulary terms (e.g., MeSH and database-specific subject headings) and free-text keywords were used. Search terms were refined through preliminary searches to ensure adequate coverage of FGM/C, physiotherapy, rehabilitation, pelvic health, continence care, chronic pain, psychosexual health, and multidisciplinary management. Detailed information regarding databases searched, search strategies, and grey literature sources is provided in the Information Sources and Search Strategy section and Supplementary File 1.

### Eligibility criteria

Eligibility criteria were developed using the Population–Concept–Context (PCC) framework recommended by the Joanna Briggs Institute for scoping reviews. The PCC framework was selected because it provides a structured approach for identifying and mapping heterogeneous evidence across diverse study designs, healthcare settings, and disciplinary perspectives. Eligibility criteria were designed to capture both direct rehabilitation evidence and contextual evidence relevant to physiotherapy and rehabilitation for survivors of female genital mutilation/cutting (FGM/C).

### Population

Eligible participants comprised adolescent girls (≥ 15 years) and adult women living with any World Health Organization (WHO) type of female genital mutilation/cutting (FGM/C), irrespective of ethnicity, migration status, or geographical location. The lower age threshold of 15 years was selected because rehabilitation-related outcomes of interest, including pelvic floor dysfunction, psychosexual concerns, continence problems, and functional impairments, are more likely to be reported during late adolescence and adulthood. Although adolescents were eligible for inclusion, the identified evidence predominantly involved adult women. Studies exclusively involving children younger than 15 years were excluded unless data relating to eligible participants could be extracted separately.

Studies involving healthcare professionals, physiotherapists, multidisciplinary rehabilitation teams, caregivers, and other service providers were also eligible if they provided evidence relevant to rehabilitation needs, physiotherapy practice, rehabilitation pathways, service delivery, referral processes, or multidisciplinary management for women living with FGM/C. These studies were included because the review aimed not only to synthesise evidence on rehabilitation outcomes among survivors but also to map the organisation, delivery, and implementation of rehabilitation services supporting FGM/C care.

### Concept

The concept of interest was physiotherapy and rehabilitation in relation to the management of functional impairments, symptoms, and health-related consequences associated with female genital mutilation/cutting (FGM/C). For this review, rehabilitation was broadly defined as interventions, services, care pathways, professional practices, and multidisciplinary approaches intended to optimise functioning, reduce symptom burden, enhance participation, and improve quality of life among survivors of FGM/C.

Eligible evidence included both direct rehabilitation evidence and contextual rehabilitation evidence. Direct rehabilitation evidence referred to studies that evaluated, described, implemented, or reported physiotherapy interventions, rehabilitation programmes, pelvic health interventions, continence management, chronic pain management, psychosexual rehabilitation, multidisciplinary rehabilitation services, or other rehabilitation-focused approaches involving survivors of FGM/C.

Contextual rehabilitation evidence referred to studies that did not directly evaluate rehabilitation interventions but reported findings relevant to rehabilitation practice, including functional impairments, pelvic floor dysfunction, lower urinary tract symptoms, chronic pain, psychosexual difficulties, survivor experiences, healthcare needs, service utilisation, referral pathways, access to care, and barriers to rehabilitation services. These studies were included because direct physiotherapy and rehabilitation research involving women living with FGM/C was anticipated to be limited. Consequently, contextual evidence was used to inform understanding of rehabilitation needs, service development, and future research priorities rather than to determine intervention effectiveness.

Because terminology relating to rehabilitation and physiotherapy varied considerably across the included literature, broad operational definitions of rehabilitation-related care were applied. Studies describing pelvic health physiotherapy, continence rehabilitation, psychosexual rehabilitation involving physiotherapy input, conservative pelvic floor management, chronic pain rehabilitation, or multidisciplinary rehabilitation pathways were considered eligible where rehabilitation-focused management was explicitly described.

Studies focusing exclusively on surgical management, obstetric outcomes, prevalence estimates, legal issues, policy discussions, or cultural aspects of FGM/C without relevance to rehabilitation, functioning, symptom management, healthcare service delivery, or rehabilitation needs were excluded.

### Context

The review considered evidence from all geographical regions and healthcare settings in which survivors of female genital mutilation/cutting (FGM/C) may access care, including primary care, specialist pelvic health services, rehabilitation services, community-based programmes, hospital settings, academic institutions, and multidisciplinary clinics. Studies conducted in both high-income and low- and middle-income countries were eligible for inclusion.

Given increasing global migration and the international distribution of FGM/C survivors, no restrictions were placed on geographical location, healthcare system, or service delivery model. The review sought to capture evidence relating to rehabilitation needs, service provision, care pathways, and physiotherapy practice across diverse cultural, socioeconomic, and healthcare contexts.

### Types of sources and eligibility criteria

Consistent with scoping review methodology, this review considered a broad range of evidence sources to comprehensively map the available literature on physiotherapy and rehabilitation for survivors of FGM/C. Eligible evidence sources included quantitative, qualitative, and mixed-methods studies, observational and interventional studies, case reports, case series, programme and service evaluations, clinical audits, and relevant grey literature. Clinical practice guidelines, consensus statements, policy documents, professional reports, and other practice-related documents were also eligible where they provided information relevant to physiotherapy, rehabilitation, service delivery, or multidisciplinary management of FGM/C.

Studies were eligible if they reported either direct rehabilitation evidence or contextual evidence relevant to rehabilitation practice, rehabilitation needs, rehabilitation pathways, rehabilitation-related impairments, symptoms, healthcare experiences, service delivery, referral pathways, or barriers and facilitators to rehabilitation for eligible populations. Only studies published in English and available as full-text publications were included.

Studies were excluded if they focused exclusively on children younger than 15 years without separately extractable data for eligible participants; addressed only prevalence, epidemiology, legal issues, policy debates, cultural practices, advocacy activities, or prevention initiatives without relevance to rehabilitation, functioning, symptom management, healthcare service delivery, or rehabilitation needs; focused exclusively on surgical or obstetric outcomes without rehabilitation implications; or were conference abstracts, editorials, commentaries, letters, study protocols, or publications lacking primary data or relevant rehabilitation content. Studies not published in English or unavailable as full-text publications were also excluded.

### Information sources and search strategy

A comprehensive search was undertaken to identify published and relevant grey literature relating to physiotherapy and rehabilitation for survivors of female genital mutilation/cutting (FGM/C). Electronic database searches were conducted in PubMed/MEDLINE, Embase, CINAHL, Scopus, Web of Science, and PsycINFO from database inception to the date of the final search. These databases were selected to ensure comprehensive coverage of medical, rehabilitation, nursing, allied health, psychological, and interdisciplinary literature.

The search strategy was developed using the Population–Concept–Context (PCC) framework and incorporated both controlled vocabulary terms (e.g., Medical Subject Headings [MeSH] and database-specific subject headings) and free-text keywords. Search concepts included female genital mutilation/cutting, physiotherapy, physical therapy, rehabilitation, pelvic health, continence care, chronic pain, psychosexual health, and multidisciplinary management. Search strategies were adapted as appropriate for each database.

No geographical restrictions were applied. Controlled vocabulary terms and free-text keywords were combined using Boolean operators (AND/OR) to maximise search sensitivity and retrieval of relevant studies. Language restrictions were applied during the screening stage, with only English-language publications considered eligible for inclusion.

Grey literature searching was conducted using Google Scholar. To ensure feasibility while maintaining broad coverage, the first 500 results sorted by relevance were screened for potentially eligible records. This approach has been recommended in systematic and scoping reviews to balance comprehensiveness with the practical challenges of screening very large numbers of Google Scholar records.

To enhance comprehensiveness, the reference lists of all included studies and relevant review articles were manually screened to identify additional potentially eligible publications not retrieved through the electronic searches. Detailed search strategies for all databases, including search terms, search limits, and dates of execution, are provided in Supplementary File 1.

### Study selection

Citations retrieved from the electronic database and grey literature searches were imported into Covidence systematic review software for reference management, duplicate removal, and study screening. Prior to formal screening, a pilot screening exercise involving approximately 50 titles and abstracts was undertaken by the review team to ensure consistency in study selection procedures and reviewer calibration. Any discrepancies identified during piloting were discussed and resolved before commencement of formal screening.

Two reviewers (KF and OA) independently screened the titles, abstracts, and full-text articles against the predefined eligibility criteria. Any disagreements were resolved through discussion and consensus. Where consensus could not be reached, a third reviewer (JE) acted as the adjudicator.

The study selection process was documented using a PRISMA-ScR flow diagram (Fig. 1), including the number of records identified, duplicates removed, records screened, full-text articles assessed for eligibility, reasons for exclusion, and studies included in the final synthesis (Fig. 1).

**Fig. 1:**
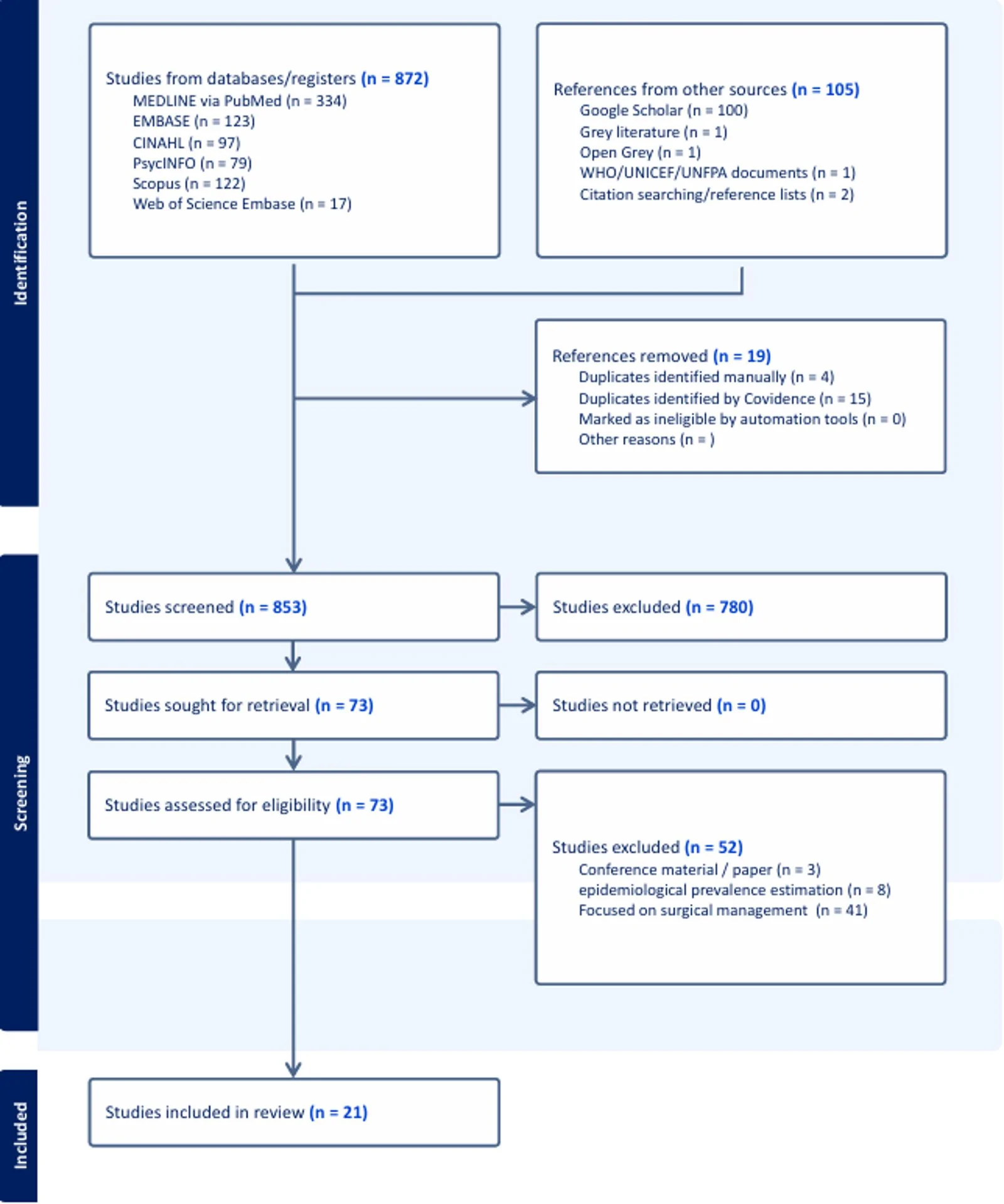
PRISMA Flow Diagram

### Data charting

A standardised data charting form was developed by the review team and piloted on a sample of included studies to ensure consistency and completeness of data charting. The charting form was refined iteratively throughout the review process, where necessary, to accommodate the breadth and diversity of the available evidence.

Data charting was undertaken independently by two reviewers (KF and OA), with discrepancies resolved through discussion and consensus. Where necessary, a third reviewer (JE) was consulted to resolve disagreements.

The following information was charted from each included source: author(s), year of publication, country of study, study aims, study design, participant characteristics, FGM/C type (where reported), healthcare setting, rehabilitation-related impairments or symptoms, physiotherapy and rehabilitation interventions, multidisciplinary care components, outcome measures, key findings, and recommendations relevant to physiotherapy and rehabilitation practice.

Additional information relating to service delivery models, referral pathways, barriers and facilitators to care, implementation considerations, and evidence gaps was charted where available. The charted data were reviewed throughout the review process to ensure consistency with the review objectives.

### Data items

Variables extracted from the included studies were aligned with the objectives of the review and included bibliographic information (author(s), year of publication, title, and country of study), study characteristics (study design, healthcare setting, study aims, and methodological approach), and participant characteristics (sample size, age, sex, migration status, and type of female genital mutilation/cutting [FGM/C], where reported).

Additional extracted variables included rehabilitation domain, type of physiotherapy or rehabilitation intervention, rehabilitation-related complications or functional impairments addressed, the role of physiotherapy within multidisciplinary care pathways, outcome measures and validated assessment instruments, implementation characteristics, barriers and facilitators to rehabilitation delivery, and implications for physiotherapy practice and future research.

Examples of rehabilitation-related outcomes extracted included chronic pelvic pain, dyspareunia, pelvic floor dysfunction, urinary incontinence, lower urinary tract symptoms, psychosexual dysfunction, pelvic organ prolapse, chronic vulvar pain, and quality-of-life indicators. Where available, validated outcome measures relating to pelvic floor symptoms, psychosexual functioning, continence outcomes, pain severity, and quality of life were also charted.

### Data analysis and synthesis

Consistent with scoping review methodology [27, 28], the extracted data were synthesised descriptively and narratively to map the breadth, characteristics, and nature of the available evidence. Descriptive statistics were used, where appropriate, to summarise study characteristics, and findings were presented in both tabular and narrative formats.

Included studies were categorised according to study design, geographical location, participant characteristics, rehabilitation-related impairments, physiotherapy and rehabilitation interventions, outcome measures, and key findings. To facilitate interpretation of the evidence base, studies were further classified as either direct rehabilitation evidence or contextual rehabilitation evidence. Direct rehabilitation evidence comprised studies that evaluated, described, or implemented physiotherapy interventions, rehabilitation programmes, or rehabilitation-focused care pathways involving survivors of FGM/C. Contextual rehabilitation evidence comprised studies that informed understanding of rehabilitation needs, functional impairments, service provision, healthcare experiences, and multidisciplinary management without directly evaluating rehabilitation interventions. This classification was developed by the review team to facilitate interpretation of a heterogeneous evidence base and was not intended to represent a hierarchy of evidence.

A narrative synthesis was undertaken to identify and summarise key themes across the included studies. Findings were organised according to rehabilitation-related impairments, physiotherapy and rehabilitation interventions, multidisciplinary care approaches, outcome measures, service delivery considerations, and evidence gaps. Owing to substantial heterogeneity in study designs, participant characteristics, interventions, outcome measures, and reported outcomes, quantitative synthesis (meta-analysis) was not appropriate [27–29].

## Result

### Characteristics of included studies

Of the 872 records identified through database and grey literature searches, 21 studies met the inclusion criteria and were included in this scoping review (Fig. 1). The included studies were conducted across diverse geographical settings. Switzerland contributed the highest number of studies (*n* = 5), followed by the United Kingdom and Egypt (*n* = 3 each). Sudan and the United States each contributed two studies, while Kenya and Türkiye contributed one study each. Three studies comprised international or multinational guidance and implementation frameworks. Overall, the evidence base was concentrated in high-income countries and specialist migrant health service settings.

Most included studies (18/21; 85.7%) were published between 2016 and 2025, reflecting increasing research attention to the long-term rehabilitation, psychosexual, pelvic health, and urogynaecological consequences of FGM/C.

The included studies demonstrated substantial methodological heterogeneity. Study designs comprised cross-sectional studies (8 studies; 38.1%), narrative reviews and clinical guidance papers (4 studies; 19.0%), qualitative studies (2 studies; 9.5%), case reports (2 studies; 9.5%), and one study each (4.8%) for retrospective cohort, randomized controlled trial, survey, case-control, and systematic/scoping review designs. The available evidence was predominantly descriptive and observational in nature, with only one randomized controlled trial and one rehabilitation-focused case report directly evaluating rehabilitation interventions [8, 21].

The methodological characteristics and distribution of the included evidence are summarised in Table 1. Overall, observational and descriptive studies accounted for the majority of the evidence base (15/21; 71.4%), whereas only two studies (9.5%) directly evaluated rehabilitation interventions. Narrative reviews, clinical guidance documents, and one systematic/scoping review provided additional contextual evidence relevant to physiotherapy and multidisciplinary rehabilitation. These findings highlight the emerging nature of the evidence base and the limited availability of intervention studies evaluating rehabilitation outcomes in women living with FGM/C.

**Table 1:** Methodological Characteristics and Distribution of the Included Evidence

Indicator	*n* (%)
Study design	
Randomized controlled trial	1 (4.8)
Cross-sectional studies	8 (38.1)
Qualitative studies	2 (9.5)
Case reports	2 (9.5)
Retrospective cohort study	1 (4.8)
Survey study	1 (4.8)
Case-control study	1 (4.8)
Narrative reviews/clinical guidance	4 (19.0)
Systematic/scoping review	1 (4.8)
Evidence type	
Direct rehabilitation intervention studies (RCT and case report)	2 (9.5)
Observational/descriptive studies	15 (71.4)
Population characteristics	
Studies involving adolescents (≥ 15 years)	1 (4.8)
Studies predominantly involving adult women	20 (95.2)

Consistent with the review framework, 11 studies contributed direct rehabilitation evidence, including studies evaluating or describing physiotherapy interventions, rehabilitation-focused management approaches, multidisciplinary rehabilitation pathways, or clinical rehabilitation guidance. However, only two of these studies directly evaluated rehabilitation interventions. The remaining 10 studies contributed contextual rehabilitation evidence by providing information on rehabilitation needs, psychosexual health, healthcare experiences, service delivery, implementation considerations, and barriers and facilitators to care.

### Rehabilitation-related complications and functional impairments

Findings relating to rehabilitation-related complications and functional impairments were derived from both the Core Clinical and Technical Evidence Map (Table 2) and the Contextual and Survivor-Centred Rehabilitation Matrix (Table 3). Core clinical studies primarily quantified symptom burden, functional impairment, and rehabilitation-relevant outcomes, whereas contextual studies provided insight into survivor experiences, psychosocial consequences, healthcare access, and rehabilitation needs.

**Table 2:** Core Clinical & Technical Evidence Map of Rehabilitation Needs and Interventions in Women With FGM/C

S/*N*	Author & Year	Study Design & Country	Population Characteristics	Rehabilitation-Relevant Domain	Intervention / Concept Mapped	Physiotherapy / Rehabilitation Intervention	Outcome Measures / Variables	Key Findings / Rehabilitation Outcomes
1	Broom (2017) [22]	Survey Study, United Kingdom	Women’s health physiotherapists involved in pelvic health care	Clinical Practice Mapping	Physiotherapy practice patterns and management approaches for women with FGM/C	Pelvic health physiotherapy approaches reported inconsistently across clinicians	Physiotherapy experience, treatment approaches, referral patterns	Only a minority of physiotherapists had managed FGM-related complications; rehabilitation approaches lacked standardization.
2	Abdulcadir & Dällenbach (2013) [8]	Case Report, Switzerland	27-year-old Somali migrant woman with Type III FGM/C	Urogynecological Rehabilitation	Defibulation combined with biofeedback re-educative therapy and multidisciplinary counseling	Biofeedback re-educative therapy; multidisciplinary counseling	Urgency, urge incontinence, dyspareunia, voiding symptoms	Urgency and urge incontinence resolved after rehabilitation intervention; sexual intercourse became possible and bladder symptoms improved.
3	Sakr et al. (2023) [21]	Randomized Controlled Trial, Egypt	Adult women with FGM-related sexual dysfunction	Sexual Rehabilitation	Clitoral therapy device for rehabilitation of FGM-associated sexual dysfunction	Clitoral therapy device intervention	Sexual function, psychosexual outcomes	Significant improvement in psychosexual and functional sexual outcomes following rehabilitation-focused intervention.
4	Binkova et al. (2021) [4]	Cross-sectional Study, Switzerland	124 migrant women with FGM/C	Pelvic Floor Dysfunction Mapping	Assessment of pelvic floor distress, pelvic floor impact, and psychosexual symptoms in women with FGM/C	No direct physiotherapy intervention; rehabilitation-needs mapping study	PFDI-20, PFIQ-7, sexual function, genital self-image	Women experienced clinically significant pelvic floor distress and psychosexual impairment affecting quality of life and daily functioning.
5	Birge et al. (2021) [6]	Observational Study, Sudan	Women with Type I–III FGM/C	Pelvic Organ Prolapse Mapping	Association between FGM/C type and pelvic floor dysfunction/pelvic organ prolapse	No direct physiotherapy intervention; dysfunction mapping study	Pelvic organ prolapse severity, pelvic floor dysfunction	Type III FGM/C showed strong association with symptomatic pelvic organ prolapse and severe pelvic floor dysfunction.
6	Geynisman-Tan et al. (2019) [5]	Cross-sectional Survey, United States	44 women living with FGM/C	Lower Urinary Tract Dysfunction	Assessment of lower urinary tract symptoms and pelvic floor impact in women with FGM/C	No direct physiotherapy intervention; symptom burden assessment	LUTS, urinary hesitancy, nocturia, urgency, stress incontinence, PFIQ-7	High prevalence of lower urinary tract symptoms and substantial quality-of-life impairment among women with FGM/C.
7	Elneil (2016) [7]	Clinical Narrative Review, United Kingdom	Women with FGM and female sexual dysfunction	Multidisciplinary Rehabilitation	Multidisciplinary management approaches for FGM-related sexual dysfunction and chronic pelvic pain	Pelvic floor physiotherapy; pelvic floor exercises/training; psychosexual rehabilitation; chronic pelvic pain rehabilitation	Sexual dysfunction, chronic pain, rehabilitation approaches	Highlighted importance of individualized physiotherapy, pelvic floor rehabilitation, psychological support, and multidisciplinary management.
8	Kingston et al. (2025) [10]	Narrative Literature Review	Women with FGM-related incontinence	Urogynecological Rehabilitation Needs	Narrative synthesis of FGM-associated incontinence complications and conservative management approaches	Bladder-awareness training; conservative continence management; talking therapy	LUTS, stress incontinence, urgency, prolapse, fistula, conservative treatments	Identified major urogynecological complications and substantial evidence gaps in conservative rehabilitation and continence management.
9	Bazzoun et al. (2021) [9]	Retrospective Cohort Study, Switzerland	506 women attending specialized FGM clinic	Chronic Pain Rehabilitation	Clinical characteristics of chronic vulvar pain and dyspareunia in women with FGM/C	No direct physiotherapy intervention; chronic pain characterization study	Dyspareunia, vulvar pain, scar complications, neuromas	Chronic vulvar pain and dyspareunia were highly associated with scar complications, vulvar cysts, neuromas, and Type III FGM/C.
10	WHO (2018) [1]	International Guideline	Women and girls affected by FGM/C globally	Clinical & Rehabilitation Guidance	WHO recommendations for management of health complications associated with FGM/C	Psychosexual support; continence care; multidisciplinary rehabilitation-informed management	Multidisciplinary management recommendations	Recommended multidisciplinary rehabilitation-informed management including psychosexual support, urogynecological care, and culturally sensitive services.
11	Abdulcadir et al. (2016) [12]	Cross-sectional Study, Switzerland	Women with and without FGM/C	Functional Sexual Rehabilitation Mapping	Assessment of sexual anatomy, genital tissue volume, and sexual function in women with and without FGM/C	No direct physiotherapy intervention; functional sexual outcome assessment	FSFI, sexual desire, dyspareunia, genital anatomy	Women with FGM/C demonstrated lower sexual function scores, increased dyspareunia, and altered genital tissue volume relevant to rehabilitation planning.

**Table 3:** Contextual, Implementation & Survivor-Centered Rehabilitation Matrix

S/*N*	Study	Study Type	Clinical/Contextual Relevance	Rehabilitation Translation Value	Specific Contribution to Rehabilitation Understanding
1	Anis et al. (2012) [13]	Cross-sectional Study	Sexual function among Egyptian women with FGM/C	Psychosexual rehabilitation rationale	Identified reduced arousal, orgasmic dysfunction, dyspareunia, and decreased sexual satisfaction, supporting integration of psychosexual counseling and pelvic rehabilitation into survivor care.
2	Ismail et al. (2017) [30]	Case-Control Study	Sexual dysfunction in women with Type I and II FGM/C	Pelvic rehabilitation relevance	Demonstrated that women with FGM/C types I and II had significantly poorer sexual function scores than controls, suggesting need for pelvic floor rehabilitation and chronic pain management strategies.
3	Rouzi et al. (2017) [31]	Cross-sectional Study	Sexual function among Sudanese women	Survivor-centered rehabilitation rationale	Reported persistent impairment in desire, lubrication, orgasm, and satisfaction among women with FGM/C, emphasizing long-term psychosexual rehabilitation needs.
4	Alsibiani & Rouzi (2010) [32]	Cross-sectional Study	Sexual dysfunction and quality of life	Psychosexual rehabilitation support	Demonstrated strong association between FGM/C and reduced sexual quality of life, highlighting the need for multidisciplinary rehabilitation combining psychosexual therapy and pelvic health support.
5	Esho et al. (2017) [11]	Qualitative Study	Sexual disorders and lived experiences in Kenya	Cultural and psychosocial rehabilitation insight	Revealed that women described loss of sexual pleasure (“the heat goes away”), marital distress, and emotional suffering, underscoring importance of culturally sensitive and trauma-informed rehabilitation models.
6	Evrenol Öçal et al. (2024) [14]	Qualitative Study	Healthcare access and survivor experiences	Trauma-informed care relevance	Identified barriers including stigma, embarrassment, lack of culturally competent providers, and fear of discrimination, emphasizing need for survivor-centered and culturally safe rehabilitation services.
7	Dawson et al. (2022) [2]	Review of Guidelines and Tools	Woman- and girl-centered care frameworks	Implementation science relevance	Synthesized evidence supporting trauma-informed, culturally responsive, multidisciplinary care pathways involving gynecology, psychology, physiotherapy, and social support services.
8	El Ayadi et al. (2020) [24]	Systematic Scoping Review	Reintegration after fistula surgery	Holistic rehabilitation framework	Demonstrated that rehabilitation after genital fistula surgery extends beyond physical recovery to include psychosocial rehabilitation, economic reintegration, and long-term community support.
9	Wallace et al. (2019) [16]	Narrative Review	General pelvic floor physical therapy principles	Transferable rehabilitation framework	Provided evidence supporting pelvic floor muscle training, bladder retraining, manual pelvic therapy, and behavioral interventions that may be transferable to management of FGM-related pelvic floor dysfunction.
10	Ghaderi et al. (2019) [17]	Randomized Clinical Trial	Pelvic floor rehabilitation for dyspareunia	Transferable intervention evidence	Demonstrated that pelvic floor muscle training, electrotherapy, and manual therapy significantly improved dyspareunia and pelvic floor function, offering potentially adaptable rehabilitation strategies for FGM survivors with chronic pelvic pain and sexual dysfunction.

Psychosexual dysfunction was the most frequently reported rehabilitation-related complication, identified in 12 studies (57.1%) [7, 8, 11–14, 21, 30–32]. Common manifestations included reduced sexual desire, impaired arousal, orgasmic dysfunction, decreased sexual satisfaction, altered genital self-image, emotional distress, and relationship difficulties. Qualitative and survivor-centred studies further described experiences of stigma, embarrassment, reduced self-esteem, relationship difficulties, and diminished sexual wellbeing among survivors [11, 13, 14, 30–32].

Dyspareunia and chronic pelvic pain were reported in 10 studies (47.6%) [7–9, 12, 17, 19–21, 30–32]. Chronic vulvar pain, scar-related pain, neuromas, and persistent discomfort during sexual activity were frequently described, particularly among women with Type III FGM/C [8, 9].

Pelvic floor dysfunction was reported in six studies (28.6%) [4, 6, 7, 16, 17, 21]. Reported impairments included pelvic floor distress, pelvic organ prolapse, pelvic floor muscle dysfunction, and reduced pelvic health-related quality of life. Several studies reported a greater burden of pelvic floor symptoms among women with Type III FGM/C [4, 6].

Lower urinary tract symptoms and urinary incontinence were reported in five studies (23.8%) [5, 7, 8, 10, 22]. Frequently reported symptoms included urinary urgency, urge incontinence, hesitancy, nocturia, stress urinary incontinence, and voiding dysfunction. Several studies highlighted the impact of these symptoms on daily functioning and psychosocial wellbeing [5, 8, 10].

Across both evidence streams, chronic pain, sexual dysfunction, pelvic floor symptoms, and urinary difficulties were associated with reduced quality of life, restricted social participation, and challenges in accessing healthcare services [11–14, 23]. Survivors frequently reported barriers related to cultural stigma, limited availability of culturally competent healthcare providers, fear of discrimination, and poor awareness of available support services [2, 14, 23].

Overall, the included studies identified a broad spectrum of rehabilitation-related complications affecting physical, psychosexual, urinary, and functional domains. Together, the clinical and contextual evidence demonstrated the multidimensional impact of FGM/C and highlighted the need for rehabilitation approaches that address both functional impairments and broader psychosocial consequences. These findings provided the clinical rationale for the physiotherapy, rehabilitation, and multidisciplinary care approaches reported across the included studies.

### Physiotherapy and rehabilitation interventions, outcome measures, and care pathways

Findings relating to physiotherapy and rehabilitation interventions, outcome measures, and care pathways were derived from both the Core Clinical and Technical Evidence Map (Table 2) and the Contextual and Survivor-Centred Rehabilitation Matrix (Table 3). Core clinical studies primarily described rehabilitation interventions, rehabilitation-related outcomes, and symptom management approaches, whereas contextual studies provided insight into service delivery models, multidisciplinary care pathways, implementation considerations, and survivor-centred rehabilitation needs.

Rehabilitation-related approaches identified across the literature included pelvic floor physiotherapy, pelvic floor muscle training, bladder rehabilitation, conservative continence management, psychosexual counselling, chronic pain rehabilitation, manual therapy, electrotherapy, biofeedback re-education, and multidisciplinary rehabilitation pathways [7, 8, 16, 17, 21, 22]. However, most of these approaches were described within clinical guidance documents, narrative reviews, surveys, or multidisciplinary management frameworks rather than formally evaluated in intervention studies.

Only two studies directly assessed rehabilitation-focused interventions among women living with FGM/C. Abdulcadir and Dällenbach [8] reported resolution of urgency and urge incontinence following biofeedback re-educative therapy delivered as part of multidisciplinary management for a woman with Type III FGM/C. Sakr et al. [21] reported significant improvements in sexual function following the use of a clitoral therapy device among women experiencing FGM-related sexual dysfunction. No studies evaluating physiotherapy-led rehabilitation programmes using experimental or quasi-experimental designs were identified.

Outcome measures varied substantially across studies. Frequently reported measures included the Pelvic Floor Distress Inventory (PFDI-20), Pelvic Floor Impact Questionnaire (PFIQ-7), Female Sexual Function Index (FSFI), symptom-specific urinary outcomes, pain measures, genital self-image assessments, and quality-of-life indicators [4, 5, 12, 21]. Considerable heterogeneity in outcome selection and reporting was observed, limiting comparison across studies and precluding identification of a standardised outcome framework for rehabilitation research in this population.

Contextual evidence highlighted the importance of multidisciplinary and survivor-centred models of care. Several studies described care pathways incorporating gynaecology, urogynecology, physiotherapy, psychosexual therapy, psychology, continence services, and culturally responsive support services [1, 2, 7, 8, 23, 26]. Qualitative and implementation-focused studies further emphasised the importance of trauma-informed care, culturally safe service provision, coordinated referral pathways, and integrated physical and psychosocial support for women living with FGM/C [2, 14, 23].

Contextual evidence also identified barriers to effective rehabilitation, including limited provider knowledge, stigma, cultural and communication barriers, inadequate referral pathways, and restricted access to specialist services [2, 14, 23]. Collectively, the evidence demonstrated that rehabilitation for women living with FGM/C extends beyond symptom management and requires coordinated multidisciplinary care that addresses physical, psychosexual, psychological, and social dimensions of health.

### Evidence gaps identified across the core clinical and contextual evidence base

Several important evidence gaps were identified across both the core clinical and contextual rehabilitation evidence base.

First, despite the broad range of rehabilitation-related complications reported among women living with FGM/C, there was a notable scarcity of intervention-focused research. Only one randomized controlled trial [21] and one rehabilitation-focused case report [8] directly evaluated rehabilitation interventions specifically among women with FGM/C. No studies evaluating physiotherapy-led rehabilitation programmes using experimental or quasi-experimental designs were identified.

Second, considerable heterogeneity was observed in the outcome measures used across studies. Although instruments such as the Female Sexual Function Index (FSFI), Pelvic Floor Distress Inventory (PFDI-20), and Pelvic Floor Impact Questionnaire (PFIQ-7) were reported, no standardised rehabilitation outcome framework was identified. This variability limited comparison across studies and hindered synthesis of rehabilitation outcomes.

Third, the evidence base was heavily concentrated in high-income countries and specialist migrant health settings, despite the highest prevalence of FGM/C occurring in low- and middle-income countries. Few studies examined rehabilitation service delivery, implementation strategies, or physiotherapy practice within resource-constrained settings where access to rehabilitation services may be limited.

Fourth, limited evidence was available regarding rehabilitation needs across different age groups. Although adolescents aged 15 years and older were eligible for inclusion, only one study included adolescent and young adult survivors [14]. No studies specifically evaluated adolescent rehabilitation needs, rehabilitation pathways, or age-specific rehabilitation interventions.

Finally, contextual evidence highlighted important gaps relating to service delivery, provider education, referral pathways, and access to culturally responsive rehabilitation services. Several studies reported barriers associated with stigma, limited provider knowledge, inadequate multidisciplinary coordination, and restricted access to specialist services [2, 14, 23]. However, few studies evaluated strategies to address these barriers or examined implementation outcomes relevant to rehabilitation practice.

Collectively, these findings demonstrate that the current evidence base remains predominantly descriptive and observational, with limited intervention-focused research to inform physiotherapy practice, rehabilitation service development, and implementation of survivor-centred rehabilitation programmes for women living with FGM/C.

## Discussion

This scoping review mapped the current evidence relating to physiotherapy and rehabilitation for survivors of female genital mutilation/cutting (FGM/C). The findings demonstrated that women living with FGM/C experience a broad range of rehabilitation-related impairments, including psychosexual dysfunction, chronic pelvic pain, pelvic floor dysfunction, lower urinary tract symptoms, and reduced quality of life. These findings are consistent with previous reviews and international guidance highlighting the long-term physical, psychosexual, and psychosocial consequences of FGM/C [1–3, 23, 25]. Collectively, the evidence suggests that rehabilitation needs extend beyond the management of anatomical injury to encompass functional recovery, psychosocial wellbeing, and participation in daily life.

A notable finding of this review was the mismatch between the substantial rehabilitation burden reported across the literature and the limited availability of rehabilitation-focused intervention research. Viewed through the International Classification of Functioning, Disability and Health (ICF), these impairments extend beyond body structures and functions to affect activities, participation, psychosocial wellbeing, and overall quality of life [15]. This highlights the importance of rehabilitation approaches that address functional limitations alongside survivor-centred, culturally responsive, and trauma-informed care.

### Physiotherapy and rehabilitation interventions

A key finding of this review was the limited evidence directly evaluating rehabilitation interventions for women living with FGM/C. Although several rehabilitation approaches were identified, including pelvic floor physiotherapy, bladder rehabilitation, biofeedback, psychosexual counselling, and multidisciplinary rehabilitation pathways, these approaches were predominantly described in clinical guidance documents, narrative reviews, and observational studies rather than evaluated through intervention research [7, 8, 16, 17, 21, 22]. Only one randomized controlled trial [21] and one rehabilitation-focused case report [8] directly assessed rehabilitation interventions among women living with FGM/C.

This finding reflects the broader evolution of FGM/C research, which has historically focused on prevention, obstetric outcomes, surgical management, legal and human rights issues, and psychosexual consequences rather than rehabilitation [1–3, 23, 25]. Consequently, the evidence base informing physiotherapy practice remains limited.

Evidence from the broader pelvic health rehabilitation literature demonstrates that interventions such as pelvic floor muscle training, bladder retraining, manual therapy, biofeedback, behavioural interventions, and multidisciplinary rehabilitation can improve outcomes for women with dyspareunia, chronic pelvic pain, urinary incontinence, and pelvic floor dysfunction [16, 17, 19, 20, 33]. However, the applicability of these findings to women living with FGM/C remains uncertain because of the unique anatomical changes, scar-related complications, psychosexual consequences, cultural considerations, and healthcare access barriers associated with FGM/C. Consequently, although these rehabilitation principles may be transferable to FGM/C care, their effectiveness for this population has not been established and should be interpreted cautiously. Future intervention studies are needed to evaluate the safety, feasibility, acceptability, and effectiveness of physiotherapy interventions specifically tailored to women living with FGM/C.

### Outcome measures and multidisciplinary care

The review identified substantial variation in the rehabilitation outcome measures used across studies. Frequently reported instruments included the Female Sexual Function Index (FSFI), Pelvic Floor Distress Inventory (PFDI-20), and Pelvic Floor Impact Questionnaire (PFIQ-7) [4, 5, 12, 21]. However, no standardised rehabilitation outcome framework was identified, limiting comparisons across studies and restricting synthesis of rehabilitation outcomes. Development of a core outcome set for rehabilitation research in women living with FGM/C may improve consistency across future studies.

The findings also highlighted the importance of multidisciplinary and survivor-centred care. Effective management was consistently described as involving collaboration among gynaecologists, urogynecologists, physiotherapists, psychologists, psychosexual therapists, and continence specialists [1, 2, 7, 8, 23, 26]. Qualitative studies further emphasised the importance of trauma-informed and culturally responsive care in addressing barriers related to stigma, discrimination, fear, and limited access to specialist services [2, 14, 23]. These findings reinforce international recommendations advocating integrated models of care that address the complex physical, psychological, and social consequences of FGM/C.

### Clinical implications for physiotherapy practice

The findings of this review have important implications for physiotherapy practice. Physiotherapists working in pelvic health, women’s health, urogynecology, primary care, and community rehabilitation may encounter women living with FGM/C who present with chronic pelvic pain, pelvic floor dysfunction, lower urinary tract symptoms, psychosexual concerns, and functional limitations. The available evidence supports the importance of comprehensive assessment, culturally responsive communication, trauma-informed care, and multidisciplinary collaboration when managing these complex presentations. Although direct evidence supporting specific physiotherapy interventions remains limited, established principles of pelvic health rehabilitation may provide a clinically relevant framework for individualised assessment and conservative management while higher-quality evidence specific to FGM/C continues to emerge.

### Evidence gaps

Despite the substantial rehabilitation burden associated with FGM/C, the available evidence remains limited. Most included studies were observational or descriptive, with very few intervention studies evaluating rehabilitation outcomes. Furthermore, much of the available evidence originated from high-income countries and specialist migrant health services despite the highest prevalence of FGM/C occurring in low- and middle-income countries. Limited evidence was also identified regarding adolescent rehabilitation needs, implementation strategies, rehabilitation service delivery, and long-term rehabilitation outcomes.

Collectively, these findings demonstrate that rehabilitation remains an under-researched component of FGM/C care. Future research should prioritise adequately powered intervention studies, implementation research, development of standardised rehabilitation outcome measures, and evaluations of multidisciplinary rehabilitation pathways across diverse healthcare settings, particularly in low- and middle-income countries where the burden of FGM/C is greatest.

### Strengths and limitations

To our knowledge, this is the first scoping review to comprehensively map the evidence relating specifically to physiotherapy and rehabilitation for survivors of FGM/C using established JBI methodology and PRISMA-ScR reporting guidance. Nevertheless, several limitations should be acknowledged. First, the available evidence was predominantly observational or descriptive, with only two studies directly evaluating rehabilitation interventions, limiting conclusions regarding intervention effectiveness. Second, substantial heterogeneity in study designs, participant characteristics, interventions, and outcome measures precluded quantitative synthesis of the available evidence. Third, consistent with scoping review methodology, no formal critical appraisal of included studies was undertaken; therefore, the methodological quality and certainty of the available evidence were not assessed. Finally, the review was restricted to English-language publications and may not have captured relevant evidence published in other languages.

## Conclusion

This scoping review mapped the available evidence relating to physiotherapy and rehabilitation for survivors of female genital mutilation/cutting (FGM/C). The findings demonstrate that women living with FGM/C experience a broad range of rehabilitation-related impairments affecting physical, psychosexual, urinary, and psychosocial health. Although physiotherapy and rehabilitation are increasingly recognised as important components of multidisciplinary FGM/C care, direct evidence evaluating rehabilitation interventions remains scarce, with most available studies describing rehabilitation needs, service delivery, or multidisciplinary management rather than intervention effectiveness. Consequently, the current evidence base supports the need for rehabilitation services but provides limited evidence regarding the effectiveness of specific physiotherapy interventions. Future research should prioritise well-designed rehabilitation intervention studies, implementation research, and the development of standardised rehabilitation outcome measures to strengthen the evidence base and support comprehensive, survivor-centred care for women living with FGM/C.

### Recommendations

Future research should prioritise high-quality intervention studies evaluating physiotherapy and multidisciplinary rehabilitation approaches for women living with FGM/C, particularly interventions targeting pelvic floor dysfunction, chronic pelvic pain, psychosexual dysfunction, urinary symptoms, and functional limitations. Studies should also evaluate the feasibility, acceptability, implementation, and long-term effectiveness of rehabilitation interventions across diverse healthcare settings.

The development and adoption of standardised rehabilitation outcome measures are needed to improve consistency across studies and facilitate comparison of rehabilitation outcomes. Future research should incorporate measures of functioning, participation, quality of life, and patient-reported outcomes in addition to symptom severity.

Given the complex physical, psychological, and social consequences of FGM/C, future rehabilitation research and clinical service development should prioritise trauma-informed, culturally responsive, and multidisciplinary models of care. Greater attention is also needed to rehabilitation needs across the life course, particularly among adolescents and women living in high-prevalence, resource-limited settings where access to specialist rehabilitation services remains limited.

Finally, research evaluating clinician education, workforce capacity, referral pathways, and multidisciplinary service delivery is needed to support the development, implementation, and sustainability of comprehensive rehabilitation programmes for women living with FGM/C.

## Supplementary Information

Supplementary Material 1: Search Strategy. Preferred Reporting Items for Systematic reviews and Meta-Analyses extension for Scoping Reviews (PRISMA-ScR) Checklist.

## Data Availability

All data generated or analysed during this study are included in this published article and its supplementary information files. Additional information is available from the corresponding author upon reasonable request.

## Abbreviations

FGM/C: Female Genital Mutilation/Cutting
FSFI: Female Sexual Function Index
ICF: International Classification of Functioning, Disability and Health
LUTS: Lower Urinary Tract Symptoms
OAB: Overactive Bladder
OSF: Open Science Framework
PCC: Population–Concept–Context
PFDI-20: Pelvic Floor Distress Inventory-20
PFIQ-7: Pelvic Floor Impact Questionnaire-7
PFMT: Pelvic Floor Muscle Training
POP: Pelvic Organ Prolapse
PRISMA: Preferred Reporting Items for Systematic Reviews and Meta-Analyses
PRISMA-ScR: Preferred Reporting Items for Systematic Reviews and Meta-Analyses Extension for Scoping Reviews
PT: Physiotherapy
RCT: Randomized Controlled Trial
WHO: World Health Organization
