# Supplementary material for: Physiotherapy and rehabilitation for survivors of female genital mutilation/cutting: a scoping review
Source: BMC Womens Health. 2026 Jul 18;26:520. doi: 10.1186/s12905-026-04708-4 (PMC13662993; doi:10.1186/s12905-026-04708-4)
Supplement: Supplementary file 1 [file 12905_2026_4708_MOESM1_ESM.docx]

**SEARCH STRATEGY**

**PubMed**

A comprehensive search strategy was developed using a combination of Medical Subject Headings (MeSH) and free-text terms related to female genital mutilation/cutting (FGM/C), physiotherapy, rehabilitation, pelvic health, psychosexual rehabilitation, and multidisciplinary care. The search strategy combined three core concepts: the condition (FGM/C), rehabilitation and physiotherapy-related interventions, and rehabilitation outcomes/complications. The following search strategy was used:

("female genital mutilation"[MeSH Terms] OR "female genital mutilation" OR "female genital cutting" OR FGM OR FGM/C OR infibulation) AND ("physical therapy modalities"[MeSH Terms] OR physiotherapy OR "physical therapy" OR rehabilitation OR "pelvic floor rehabilitation" OR "pelvic health physiotherapy" OR "pelvic floor muscle training" OR biofeedback OR "manual therapy" OR "psychosexual rehabilitation" OR "chronic pain rehabilitation" OR "urogynecological rehabilitation" OR continence OR "bladder retraining") AND ("sexual dysfunction, physiological"[MeSH Terms] OR dyspareunia OR "chronic pelvic pain" OR "pelvic floor dysfunction" OR "urinary incontinence" OR "lower urinary tract symptoms" OR "pelvic organ prolapse" OR psychosexual OR quality-of-life) AND ("cohort studies"[MeSH Terms] OR "case-control studies"[MeSH Terms] OR "cross-sectional studies"[MeSH Terms] OR "qualitative research"[MeSH Terms] OR observational OR trial OR review OR "mixed methods")

**MEDLINE (via EBSCOhost)**

A comprehensive search strategy was developed using a combination of Medical Subject Headings (MeSH) and free-text terms related to FGM/C, rehabilitation, physiotherapy, pelvic health, and psychosexual care. The same conceptual structure was maintained across databases to ensure consistency and reproducibility.

The following search strategy was used:

(MH "Female Genital Mutilation") OR "female genital mutilation" OR "female genital cutting" OR FGM OR FGM/C OR infibulation AND (MH "Physical Therapy Modalities") OR physiotherapy OR "physical therapy" OR rehabilitation OR "pelvic floor rehabilitation" OR "pelvic health physiotherapy" OR "pelvic floor muscle training" OR biofeedback OR "manual therapy" OR "psychosexual rehabilitation" OR "chronic pain rehabilitation" OR "urogynecological rehabilitation" OR continence OR "bladder retraining" AND (MH "Sexual Dysfunction") OR dyspareunia OR "chronic pelvic pain" OR "pelvic floor dysfunction" OR "urinary incontinence" OR "lower urinary tract symptoms" OR "pelvic organ prolapse" OR psychosexual OR quality-of-life AND (MH "Cohort Studies") OR (MH "Case Control Studies") OR (MH "Cross Sectional Studies") OR (MH "Qualitative Research") OR observational OR trial OR review OR "mixed methods"

**CINAHL (via EBSCOhost)**

The search strategy was adapted for CINAHL using CINAHL Subject Headings and equivalent free-text terms to ensure comprehensive retrieval of relevant studies. The same conceptual structure was maintained across databases to ensure consistency and reproducibility.

The following search strategy was used:

(MH "Female Genital Mutilation") OR "female genital mutilation" OR "female genital cutting" OR FGM OR FGM/C OR infibulation AND (MH "Physical Therapy") OR physiotherapy OR "physical therapy" OR rehabilitation OR "pelvic floor rehabilitation" OR "pelvic health physiotherapy" OR "pelvic floor muscle training" OR biofeedback OR "manual therapy" OR "psychosexual rehabilitation" OR "chronic pain rehabilitation" OR "urogynecological rehabilitation" OR continence OR "bladder retraining" AND (MH "Sexual Dysfunction") OR dyspareunia OR "chronic pelvic pain" OR "pelvic floor dysfunction" OR "urinary incontinence" OR "lower urinary tract symptoms" OR "pelvic organ prolapse" OR psychosexual OR quality-of-life AND (MH "Cohort Studies") OR (MH "Case Control Studies") OR (MH "Cross Sectional Studies") OR (MH "Qualitative Studies") OR observational OR trial OR review OR "mixed methods"

**Preferred Reporting Items for Systematic reviews and Meta-Analyses extension for Scoping Reviews (PRISMA-ScR) Checklist**

| **SECTION** | **ITEM** | **PRISMA-ScR CHECKLIST ITEM** | **REPORTED ON PAGE #** |
| --- | --- | --- | --- |
| **TITLE** | | | |
| Title | 1 | Identify the report as a scoping review. | Page 1 |
| **ABSTRACT** | | | |
| Structured summary | 2 | Provide a structured summary that includes (as applicable): background, objectives, eligibility criteria, sources of evidence, charting methods, results, and conclusions that relate to the review questions and objectives. | Page 2 |
| **INTRODUCTION** | | | |
| Rationale | 3 | Describe the rationale for the review in the context of what is already known. Explain why the review questions/objectives lend themselves to a scoping review approach. | Page 5 |
| Objectives | 4 | Provide an explicit statement of the questions and objectives being addressed with reference to their key elements (e.g., population or participants, concepts, and context) or other relevant key elements used to conceptualize the review questions and/or objectives. | Page 5 |
| **METHODS** | | | |
| Protocol and registration | 5 | Indicate whether a review protocol exists; state if and where it can be accessed (e.g., a Web address); and if available, provide registration information, including the registration number. | Page 6 |
| Eligibility criteria | 6 | Specify characteristics of the sources of evidence used as eligibility criteria (e.g., years considered, language, and publication status), and provide a rationale. | Page 7 |
| Information sources* | 7 | Describe all information sources in the search (e.g., databases with dates of coverage and contact with authors to identify additional sources), as well as the date the most recent search was executed. | Page 7 |
| Search | 8 | Present the full electronic search strategy for at least 1 database, including any limits used, such that it could be repeated. | Page 9 |
| Selection of sources of evidence† | 9 | State the process for selecting sources of evidence (i.e., screening and eligibility) included in the scoping review. | Page 10 |
| Data charting process‡ | 10 | Describe the methods of charting data from the included sources of evidence (e.g., calibrated forms or forms that have been tested by the team before their use, and whether data charting was done independently or in duplicate) and any processes for obtaining and confirming data from investigators. | Page 12 |
| Data items | 11 | List and define all variables for which data were sought and any assumptions and simplifications made. | Page 13 |
| Critical appraisal of individual sources of evidence§ | 12 | If done, provide a rationale for conducting a critical appraisal of included sources of evidence; describe the methods used and how this information was used in any data synthesis (if appropriate). | Not done |
| Synthesis of results | 13 | Describe the methods of handling and summarizing the data that were charted. | Page 13 |
| **RESULTS** | | | |
| Selection of sources of evidence | 14 | Give numbers of sources of evidence screened, assessed for eligibility, and included in the review, with reasons for exclusions at each stage, ideally using a flow diagram. | Page 15 |
| Characteristics of sources of evidence | 15 | For each source of evidence, present characteristics for which data were charted and provide the citations. | Page 15 |
| Critical appraisal within sources of evidence | 16 | If done, present data on critical appraisal of included sources of evidence (see item 12). | Not done |
| Results of individual sources of evidence | 17 | For each included source of evidence, present the relevant data that were charted that relate to the review questions and objectives. | Page 18 |
| Synthesis of results | 18 | Summarize and/or present the charting results as they relate to the review questions and objectives. | Page 17 |
| **DISCUSSION** | | | |
| Summary of evidence | 19 | Summarize the main results (including an overview of concepts, themes, and types of evidence available), link to the review questions and objectives, and consider the relevance to key groups. | Page 21 |
| Limitations | 20 | Discuss the limitations of the scoping review process. | Page 25 |
| Conclusions | 21 | Provide a general interpretation of the results with respect to the review questions and objectives, as well as potential implications and/or next steps. | Page 25 |
| **FUNDING** | | | |
| Funding | 22 | Describe sources of funding for the included sources of evidence, as well as sources of funding for the scoping review. Describe the role of the funders of the scoping review. | Page 28 |

JBI = Joanna Briggs Institute; PRISMA-ScR = Preferred Reporting Items for Systematic reviews and Meta-Analyses extension for Scoping Reviews.

* Where *sources of evidence* (see second footnote) are compiled from, such as bibliographic databases, social media platforms, and Web sites.

† A more inclusive/heterogeneous term used to account for the different types of evidence or data sources (e.g., quantitative and/or qualitative research, expert opinion, and policy documents) that may be eligible in a scoping review as opposed to only studies. This is not to be confused with *information sources* (see first footnote).

‡ The frameworks by Arksey and O’Malley (6) and Levac and colleagues (7) and the JBI guidance (4, 5) refer to the process of data extraction in a scoping review as data charting*.*

§ The process of systematically examining research evidence to assess its validity, results, and relevance before using it to inform a decision. This term is used for items 12 and 19 instead of "risk of bias" (which is more applicable to systematic reviews of interventions) to include and acknowledge the various sources of evidence that may be used in a scoping review (e.g., quantitative and/or qualitative research, expert opinion, and policy document).

*From:* Tricco AC, Lillie E, Zarin W, O'Brien KK, Colquhoun H, Levac D, et al. PRISMA Extension for Scoping Reviews (PRISMAScR): Checklist and Explanation. Ann Intern Med. 2018;169:467–473. [doi: 10.7326/M18-0850](http://annals.org/aim/fullarticle/2700389/prisma-extension-scoping-reviews-prisma-scr-checklist-explanation).
